# A Stretchable and Safe Polymer Electrolyte with a Protecting‐Layer Strategy for Solid‐State Lithium Metal Batteries

**DOI:** 10.1002/advs.202003241

**Published:** 2021-05-01

**Authors:** Shengzhao Zhang, Taibo Liang, Donghuang Wang, Yanjun Xu, Yongliang Cui, Jingru Li, Xiuli Wang, Xinhui Xia, Changdong Gu, Jiangping Tu

**Affiliations:** ^1^ State Key Laboratory of Silicon Materials Key Laboratory of Advanced Materials and Applications for Batteries of Zhejiang Province School of Materials Science and Engineering Zhejiang University Hangzhou 310027 China; ^2^ Zhengzhou Tobacco Research Institute of CNTC Zhengzhou 450001 China

**Keywords:** flexible battery, protecting layer, solid‐electrolyte interphase, stretchable electrolyte

## Abstract

An elastic and safe electrolyte is demanded for flexible batteries. Herein, a stretchable solid electrolyte comprised of crosslinked elastic polymer matrix, poly(vinylidene fluoride‐hexafluoropropylene) (PVDF‐HFP), and flameproof triethyl phosphate (TEP) is fabricated, which exhibits ultrahigh elongation of 450%, nonflammability and ionic conductivity above 1 mS cm^−1^. In addition, in order to improve the interface compatibility between the electrolyte and Li anode and stabilize the solid‐electrolyte interphase (SEI), a protecting layer containing poly(ethylene oxide) (PEO) is designed to effectively prevent the anode from reacting with TEP and optimize the chemical composition in SEI, leading to a tougher and more stable SEI on the anode. The LiFePO_4_/Li cells employing this double‐layer electrolyte exhibit an 85.0% capacity retention after 300 cycles at 1 C. Moreover, a flexible battery based on this solid electrolyte is fabricated, which can work in stretched, folded, and twisted conditions. This design of a stretchable double‐layer solid electrolyte provides a new concept for safe and flexible solid‐state batteries.

## Graphical abstract

A stretchable polymer electrolyte is fabricated based on resilient copolymer and poly(vinylidene fluoride‐hexafluoropropylene) (PVDF‐HFP) with ultrahigh elasticity, nonflammability, and good ionic conductivity. A protective layer containing poly(ethylene oxide) (PEO) is designed to protect the electrolyte against the anode and stabilize the solid‐electrolyte interphase (SEI) during cycling. A flexible solid‐state battery is prepared using this double‐layer electrolyte, which can light a light emitting diode (LED) bulb under different deformed conditions.

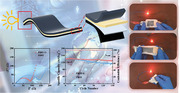

## 1 Introduction

Lithium ion batteries (LIBs) have been successfully applied in portable electronics like mobile phones and laptops in the past three decades, and are being studied for large application in electric vehicles.^[^ ^1^, ^2^, ^3^ ^]^ In addition, with the development of flexible electronics, especially wearable electronics, there is an increasing demand for reliable flexible LIBs.^[^ ^4^ ^]^ However, the thermorunaway of batteries causing burning or exploding troubles people. The volatility and flammability of liquid organic electrolyte are to be blame in these accidents.^[^ ^5^, ^6^ ^]^ The electrolytes of flexible batteries are under greater pressure because they always work in complex conditions such as folding, twisting, and stretching, which may trigger thermorunaway more easily. Thereby, it is called for safer electrolyte for flexible LIBs.

Solid electrolyte as the substitute of liquid electrolyte owns nonvolatility, low flammability, and high electrochemical and thermal stability,^[^ ^7^, ^8^, ^9^ ^]^ the employing of which in flexible LIBs will enhance the safety dramatically. At the same time, solid electrolyte is required to be elastic and deformable to keep against damage during deformation.^[^ ^10^ ^]^ Conventional inorganic solid electrolytes are too rigid to be out of shape, which cannot be used as the electrolytes for flexible batteries.^[^ ^11^, ^12^ ^]^ Meanwhile, organic electrolytes (e.g., solid polymer electrolytes (SPEs) and gel polymer electrolytes (GPEs)) based on flexible polymers are competent to work under deformed conditions.^[^ ^13^, ^14^ ^]^ SPEs always suffer from low ionic conductivity and relative poor interfacial compatibility with electrodes.^[^ ^15^, ^16^ ^]^ By comparison, GPEs deliver better electrochemical performance than SPEs due to the addition of liquid state plasticizers.^[^ ^13^, ^17^ ^]^ Nevertheless, the polymer matrix and plasticizer of GPE need to be ingeniously designed in order to be applied in safe flexible batteries.

Polymer matrix is the key to realize elasticity of GPE. At present, most flexible batteries reported can be bent and twisted, but cannot be stretched due to the lack of elasticity of electrodes and electrolytes,^[^ ^18^, ^19^, ^20^ ^]^ which restricts their applications in wearable electronics and special equipment. It is mainly because the polymers chosen as the matrix such as PEO,^[^ ^21^, ^22^ ^]^ PVDF,^[^ ^23^, ^24^ ^]^ and polycarbonate^[^ ^25^, ^26^ ^]^ have little elasticity. Therefore, there is a need for resilient polymer matrix like rubber, polysiloxane,^[^ ^27^ ^]^ or polyacylate.^[^ ^28^ ^]^ On the other hand, the plasticizer as a critical component influences the chemical and electrochemical properties of GPE, which must be nonflammable and less volatile to ensure the safety.^[^ ^29^ ^]^ Thus, some common flameproof solvents like triethyl phosphate (TEP)^[^ ^30^, ^31^, ^32^ ^]^ and fluoroethylene carbonate (FEC)^[^ ^33^, ^34^ ^]^ are able to be chosen as the plasticizers. At the same time, both the polymer matrix and plasticizer are expected to work with high‐voltage cathode materials.

In view of this, we creatively fabricate a flexible GPE (named as PBPF) consisting of a copolymer of butyl acrylate (BA) and poly‐(ethylene glycol) diacrylate (PEGDA), an analogue of rubber, as the polymer matrix,^[^ ^35^, ^36^ ^]^ PVDF‐HFP as the reinforcing agent,^[^ ^37^, ^38^ ^]^ and flameproof TEP and FEC. The PBPF electrolyte exhibits good fire resistance, outstanding mechanical properties, and good ionic conductivity. However, TEP has a poor compatibility with anodes such as lithium and graphite,^[^ ^31^, ^39^ ^]^ leading to the instability of PBPF when touched with anodes. Thus, a protecting layer is prepared onto the side where PBPF is in contact with anode. This layer (named as PBPO) is comprised of BA‐PEGDA‐PEO matrix and EC/DMC plasticizer. The PBPO electrolyte layer can effectively protect the anode from contacting with TEP in the PBPF and stabilize the SEI during cycling. Therefore, the LFP/Li cells incorporated with the double‐layer electrolyte (PBPF‐O) exhibit improved and more stable electrochemical performance. More importantly, a flexible LIB was successfully fabricated using this stretchable electrolyte by an in situ UV‐curing method where the cathode and anode were both resilient. The flexible battery can light a light emitting diode (LED) bulb under stretched, folded, and twisted condition. The design of the stretchable double‐layer GPE promotes the practical application of flexible solid‐state batteries.

## 2 Results and Discussion

**Figure 1:**
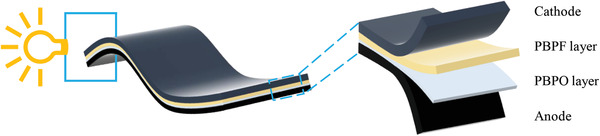
Schematic illustration of the stretchable battery based on flexible PBPF‐O electrolyte and flexible electrodes.

**Figure 2:**
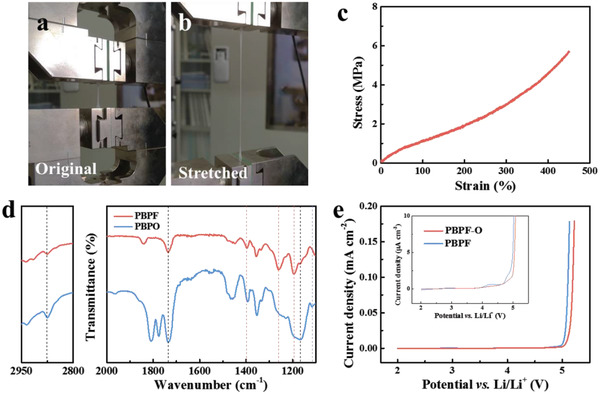
a,b) Tensile tests of PBPF membrane; c) stress–strain curve of PBPF membrane; d) FTIR spectra of PBPF and PBPO; e) LSV curves of PBPF‐O and PBPF from 2.0 to 5.5 V (vs Li/Li^+^) at 0.1 mV cm^−1^. (the insert is the magnified figure.)

The design of the double‐layer polymer electrolyte and flexible solid‐state battery is illustrated in **Figure** **1**, where the PBPF electrolyte membrane is fabricated using a facile UV‐curving method. The precursor gel containing BA monomer, PEGDA, PVDF‐HFP, LiTFSI, and mixed plasticizer of TEP and FEC is coated on the substrate and solidified under UV light. The crosslinked BA and PEGDA endow the electrolyte with viscoelasticity and PVDF‐HFP improves the strength of the membrane at the same time. Consequently, the hybrid electrolyte possesses ultrahigh elasticity with strain up to 450% as shown in **Figure** **2**a–c, ensuring its application in flexible solid‐state batteries under stretched condition. In addition, the burning test of PBPF is carried out as exhibited in Figure S1 in the Supporting Information. The PBPF membrane is not lit after touched with fire and just scorched, revealing fire resistance owing to the presence of TEP and FEC.

Nevertheless, considering that the plasticizer TEP has high reactivity to anode, another PBPO solid electrolyte layer is designed in order to avoid the direct contact between PBPF and anode. Among the components of PBPO, the mixed plasticizers EC/DMC are instead of TEP due to their stability against Li metal. Moreover, PEO takes the place of PVDF‐HFP because it is more compatible with EC/DMC. It is noted that the strength of PBPO is compromised since PEO substitutes for PVDF‐HFP in electrolyte membrane. The double‐layer electrolyte maintains satisfactory elasticity and strength owing to the robust PBPF and viscoelastic PBPO (Figures S2 and S3, Supporting Information). In addition, the burning test of PBPF‐O is carried out as well. It is observed from Figure S4 in the Supporting Information that PBPF‐O is partly lighted after touched with fire due to the flammability of PBPO (containing flammable EC/DMC). Whereas, PBPF‐O is self‐extinguished soon owing to the existence of TEP/FEC and relatively small proportion of PBPO to PBPF‐O. The PBPF‐O electrolyte membrane still possesses relative safety.

Fourier infrared spectroscopy (FTIR) is measured to identify the functional groups of PBPF and PBPO. As shown in Figure 2d, the peak at about 1402 cm^−1^ is caused by —CH_2_— bonds. The adsorption peak at around 1263 cm^−1^ is assigned to the vibration of P=O belonging to TEP.^[^ ^40^ ^]^ The adsorption peak at ≈1188.1 cm^−1^ is related to the —CF_2_ vibration demonstrating the existence of PVDF‐HFP.^[^ ^40^ ^]^ In addition, the peaks at 1720 and 1163 cm^−1^ are assigned to the C=O vibration and antisymmetric C—O stretching respectively, which verifies the acrylate group existed in BA and PEGDA.^[^ ^41^ ^]^ Moreover, the PEO component in PBPO can be verified by the peak at ≈1107 cm^−1^.^[^ ^42^ ^]^ The existence of LiTFSI in the electrolyte is demonstrated by the peak at around 2880 cm^−1^ due to the S—H_3_ stretching.^[^ ^42^ ^]^ Thermogravimetric analysis (TGA) was performed to evaluate the thermal stability of the electrolyte membranes. The TGA curves of PBPF and PBPO are shown in Figure S5 in the Supporting Information. It is noticed that the plasticizer in PBPF and PBPO begins to volatilize obviously when the temperature exceeds ≈100 and ≈120 °C, respectively. And the decomposition temperature of the polymer matrix is about 330 and 340 °C for PBPF and PBPO, respectively. The PBPF‐O electrolyte can normally work below 100 °C.

In addition to excellent mechanical properties of PBPF‐O, this composite electrolyte also exhibits satisfactory electrochemical performance. The temperature‐dependent Li^+^ conductivities of PBPF electrolyte are shown in Figure S6 in the Supporting Information. The PBPF electrolyte exhibits a room‐temperature ionic conductivity up to 1.8 mS cm^−1^. Meanwhile, the PBPO layer also has an ionic conductivity above 1 mS cm^−1^ (Figure S7, Supporting Information). The ionic conduction of the double‐layer electrolyte meets the needs of flexible solid‐state batteries. The linear sweep voltammetry (LSV) curves exhibit the electrochemical window of the PBPF and PBPF‐O electrolytes (Figure 2e). It is seen that the decomposition potential of PBPF and PBPF‐O is above 4 V (vs Li/Li^+^), satisfying the basic demand for commercial electrode materials such as LiFePO_4_.

**Figure 3:**
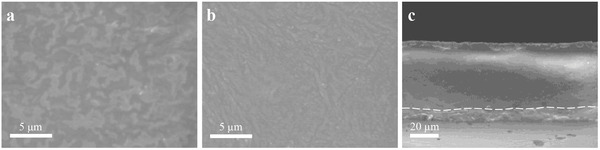
Surface SEM images of a) PBPF layer, b) PBPO layer, and c) cross‐section SEM image of PBPF‐O electrolyte membrane.

**Figure** **3**a,b shows the surface SEM images of PBPF, PBPO layer of PBPF‐O, respectively. It is noticed that wrinkles are evenly distributed on the surface of PBPF and PBPO, which are caused by the polymerizing process of BA and PEGDA during UV curing. In addition, it is seen from the cross‐section image of PBPF‐O that the thickness of PBPF and PBPO are ≈45 and ≈7 µm (Figure 3c; Figure S8, Supporting Information), respectively. It is worth mentioning that the PBPO as a functional layer is just aimed to protect the anode from PBPF. It is fabricated as thin as possible to keep the mechanical properties of PBPF‐O due to its relatively low strength. Whereas, this thin PBPO layer improves the interface compatibility between the electrolyte and Li metal and helps construct a stable SEI on the anode. The specific mechanism will be discussed in detail later.

**Figure 4:**
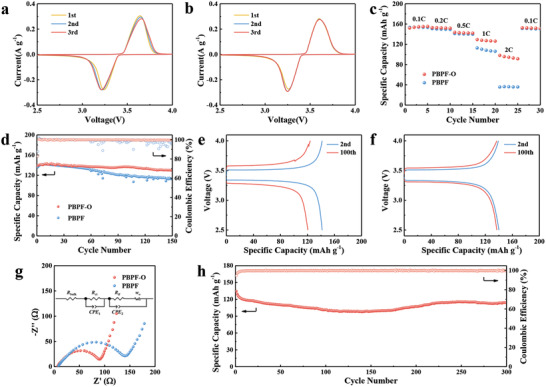
CV curves of a) LFP/PBPF/Li and b) LFP/PBPF‐O/Li cells at a scan rate of 0.1 mV s^‒1^ between 2.5 and 4.0 V (vs Li/Li^+^). Electrochemical performances of LFP/PBPF/Li and LFP/PBPF‐O/Li cells: c) rate capability; d) cycle performance at 0.5 C; g) Nyquist plots before cycling. (the inset is the equivalent circuit.) Galvanostatic charge/discharge profiles of e) LFP/PBPF/Li and f) LFP/PBPF‐O/Li cells at 0.5 C. h) Cycle performance of LFP/PBPF‐O/Li cell at 1 C.

In order to demonstrate the roles the PBPO layer plays, Li metal is chosen as the anode and the LFP/PBPF/Li and LFP/PBPF‐O/Li coin cells are assembled. The cyclic voltammetry (CV) curves of both the cells are measured at a scanning rate of 0.1 mV s^−1^ as shown in **Figure** **4**a,b, respectively. Although the LFP/PBPF/Li cell has similar cathodic and anodic peaks as LFP/PBPF‐O/Li (3.24 and 3.64 V vs 3.26 and 3.60 V, respectively) at the first cycle, its polarization increases obviously during the second and the third cycles. By comparison, the CV curves of LFP/PBPF‐O/Li remain stable during the first three cycles. This is probably attributed to the more stable interface of PBPF‐O/Li than PBPF/Li. The rate capabilities of the cells are tested from 0.1 C to 2 C (Figure 4c, corresponding galvanostatic charge/discharge profiles are shown in Figure S9 in the Supporting Information). Both the cells exhibit similar discharge capacities at 0.1 C (153.8 mAh g^‒1^ for LFP/PBPF/Li and 153.6 mAh g^‒1^ for LFP/PBPF‐O/Li). However, the capacity of LFP/PBPF/Li cell drops quickly and the gap with LFP/PBPF‐O/Li is getting larger with the increase of current density. The LFP/PBPF/Li cell only delivers 36.4 mAh g^‒1^ at 2 C while its counterpart can still deliver 95.7 mAh g^‒1^. In addition to the difference of rate capability, the comparison of cycling performance at 0.5 C between two cells is also carried out. As exhibited in Figure 4d, the LFP/PBPF‐O/Li cell remains 129.8 mAh g^‒1^ (93.0% capacity retention) after 150 cycles. At the same time, its Coulombic efficiency keeps above 99.5% during cycling. Meanwhile, the capacity retention of LFP/PBPF/Li is only 113.4 mAh g^‒1^ (83.4%). Moreover, the Coulombic efficiency of LFP/PBPF/Li begins to decrease significantly after the 60th cycle and the capacity drops together with it, which means the instability of charging and discharging process. The galvanostatic charge/discharge profiles of LFP/PBPF/Li and LFP/PBPF‐O/Li cells are presented in Figure 4e,f, respectively. The results are consistent with cyclic capacities in Figure 4d. In addition to the capacity fading and the polarization increasing, it is observed that several sharp peaks appear on the charging curve of LFP/PBPF/Li at the 100th cycle. By comparison, the charge/discharge curves of LFP/PBPF‐O/Li remain stable and the increment of polarization is tiny. We can draw an inference from Figure 4d–f that an unstable SEI formed at the PBPF/Li interface during cycling and it cracked with fresh Li metal exposed in the subsequent process, which caused numerous Li ions from electrolyte gathering at the interface and forming a new SEI during charging. This process happens irregularly, leading to the increased polarization and decreased capacity.

The electrochemical impedance spectroscopy (EIS) is measured as exhibited in Figure 4g. The resistance of charge transfer (*R*_ct_) is represented by the semicircle in the high frequency region,^[^ ^43^ ^]^ which is ≈132 Ω for LFP/PBPF/Li and ≈82 Ω for LFP/PBPF‐O/Li, respectively. The cell with PBPF‐O electrolyte has smaller resistance, benefiting for the migration of ions. In addition, the EIS of both cells after cycling are shown in Figure S10 in the Supporting Information. The resistance of LFP/PBPF‐O/Li is much smaller than that of LFP/PBPF/Li as well, indicating that a more stable SEI during cycling can be speculated. Although both the cells exhibit similar electrochemical performance at the beginning of cycling or at lower current density, the lack of PBPO layer makes the PBPF perform worse when more ions react with the Li anode at the interface during cycling. The PBPO layer indeed improves the electrochemical performance of PBPF‐O electrolyte. Finally, the cycling performance of LFP/PBPF‐O/Li cell at 1 C is carried out to further explore its potential (Figure 4h), which keeps 114.3 mAh g^‒1^ with 85.0% capacity retention after 300 cycles.

**Figure 5:**
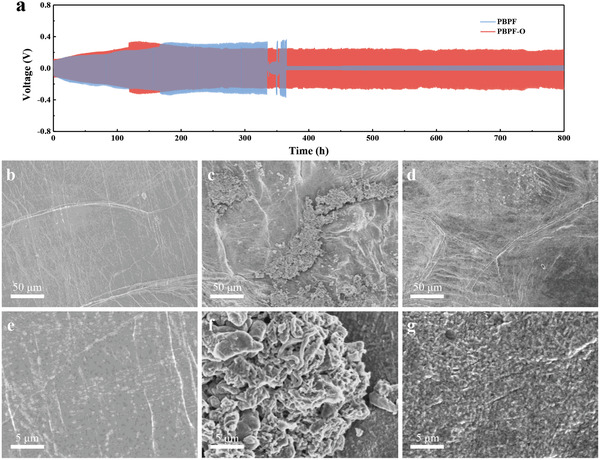
a) Galvanostatic charge/discharge voltage profiles of Li/PBPF/Li and Li/PBPF‐O/Li cells at 0.2 mA cm^‒2^. Surface SEM images of lithium anode b,e) before cycling, c,f) after 150 cycles at 0.5 C from LFP/PBPF/Li cell, and d,g) after 150 cycles at 0.5 C from LFP/PBPF‐O/Li cell, respectively.

In order to deeply understand the mechanism of PBPO layer acting on the PBPF‐O electrolyte, Li/PBPF/Li and Li/PBPB‐O/Li symmetric cells are assembled and galvanostatic charge/discharge tests are performed at a current density of 0.2 mA cm^‒2^ where each charge/discharge step lasts for 1 h. As shown in **Figure** **5**a, the overpotentials of the cells increase gradually in the initial stage from ≈0.1 to ≈0.3 V. This process is companied with the construction of SEI. The overpotential of Li/PBPB‐O/Li reaches the highest value at around 130 h and starts to decrease and finally stabilizes at 0.21–0.24 V from 200 h until 800 h, while the overpotential of Li/PBPF/Li comes to the highest value at about 180 h without a decreasing stage and becomes unstable since 334 h until short circuit happens (362 h). These differences can also be ascribed to the PBPO layer influencing the forming process of SEI and stabilizing it.

Furthermore, the morphologies of SEI on the surface of Li anodes from LFP/PBPF/Li and LFP/PBPF‐O/Li cells after 150 cycles at 0.5 C are observed. Compared with the anodes before cycling (Figure 5b,e), it is seen from Figure 5c that there are many cracks on the SEI where numerous weed‐like lithium dendrites grow on the Li anode in LFP/PBPF/Li (Figure 5f). In contrast, Figure 5d,g presents a flat and smooth SEI on the Li anode in LFP/PBPF‐O/Li cell. Even though it seems that there are several cracks, no lithium dendrites grow from them. The magnified image of the cracks in Figure 5d is shown in Figure S11 in the Supporting Information. It indicates that the SEI formed with the aid of PBPO is more intact, which effectively suppresses the growth of lithium dendrite without cracking.

**Figure 6:**
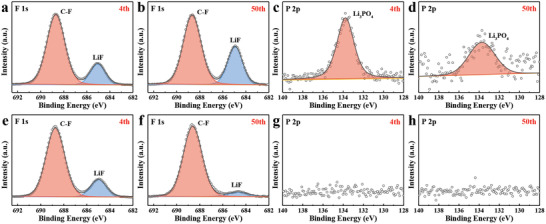
XPS spectra of Li anodes a–d) from LFP/PBPF/Li and e–h) from LFP/PBPF‐O/Li cells after different cycles at 0.5 C, respectively.

X‐ray photoelectron spectroscopy (XPS) is applied to probe the chemical composition of the SEI on Li anodes so as to explore the reason why better SEI is constructed in the presence of PBPO. First, the F 1s spectra of Li anodes in LFP/PBPF/Li and LFP/PBPF‐O/Li cells at different stages of cycling are presented in **Figure** **6**a,b and Figure 6e,f, respectively. It is seen that the ratio of LiF to C‐F organics of LFP/PBPF/Li is slightly higher than that of LFP/PBPF‐O/Li after 4 cycles. This may be ascribed to the more FEC in PBPF reacting with lithium metal. However, the LiF/C‐F ratio of LFP/PBPF/Li becomes high after 50 cycles, while the LiF/C‐F gets much low for the Li anode in LFP/PBPF‐O/Li. Although LiF is always regarded as a beneficial product in the SEI,^[^ ^44^, ^45^ ^]^ too much inorganic component may lead to the brittleness of the SEI. Furthermore, the low ionic conductivity of LiF may lead to high resistance of the SEI.^[^ ^46^ ^]^ Then, the P 2p spectra of the two anodes are measured as shown in Figure 6c,d,g,h, respectively. For the LFP/PBPF/Li cell, the decomposition product Li_3_PO_4_ of TEP is detected on the surface of Li anode. In addition, differet from the beginning stage, its peak intensity gets weaker when cycled for 50 loops, which is caused by the substitution of LiF for Li_3_PO_4_.^[^ ^40^ ^]^ And this also accounts for the increasing of LiF component during cycling for the LFP/PBPF/Li cell. Whereas, Li_3_PO_4_ can hardly be dectected on the surface of the Li anode in LFP/PBPF‐O/Li, which demonstrates the effect of PBPO layer on restraining the reaction between TEP and lithium metal. In addition, the elemental proportions in the SEI of the cells at different stage of cycling are compared as well (Figure S12, Supporting Information). The significant increase of C elecment and the decrease of Li with cycling in the PBPF‐O sample further prove the growing proportion of organic components in the SEI, which is much higher than that in PBPF. Therefore, it can be made a summary that on the one hand the PBPO layer prevents the reaction between active TEP and lithium metal. On the other hand, it increases the ratio of organic component in the SEI with increasing the number of cycles, which makes the SEI tougher during cycling. This can explain why the overpential decreases in the Li/PBPF‐O/Li cells during cycling. The inference we made above that accouts for the more stable performance of LFP/PBPF‐O/Li duing charging/discharging is also verified.

**Figure 7:**
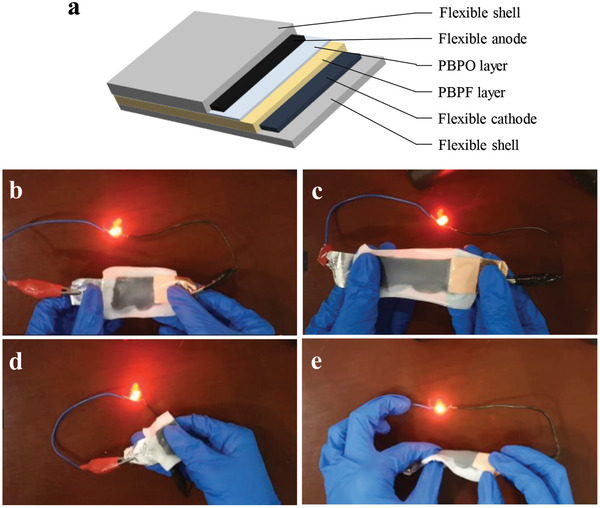
a) Schematic illustration of the design of stretchable Li‐ion battery. Flexible solid‐state battery under a) original, b) stretched, c) folded, and d) twisted condition.

Finally, a flexible solid‐state LFP/graphite battery is designed as illustrated in **Figure** **7**a. The flexible polymer BA‐PEGDA‐PVDF‐HFP which is the matrix of PBPF is applied as the shell of the battery. The elastic conductive substrate consisting of BA‐PEGDA, PVDF‐HFP and CNTs is coatd on the flexible shell as the current collector where the LFP cathode is coated. Then the PBPF‐O electrolyte is coated on the cathode followed by covering of the graphite anode which is prepared using the same procedures as the cathode. Surprisingly, the fabrication process does not need extra encapsulation procedure because of the viscoelasticity of the BA‐PEGDA‐PVDF‐HFP polymer. This flexible solid‐state battery successfully lights the LED bulb under stretched, folded, twisted condition as shown in Figure 7b–e, demonstrating the potential of the resilient polymer electrolyte.

## 3 Conclusion

In this work, we fabricated a hybrid polymer electrolyte composed of elastic polymerized BA‐PEGDA, fireproof TEP and FEC, and PVDF‐HFP, which exhibits ultrahigh resilience with elongation up to 450%, good flame resistance, and satisfactory electrochemical properties. In order to prevent the TEP component from reacting with anode, a thin buffer layer consisting PEO and EC/DMC is designed. This PBPO layer cuts off TEP and anode to help create a tough and stable SEI with more organic component without phosphate during cycling, which effectively blocks the growth of lithium dendrite and reduces the resistance of SEI. As a result, the LFP/PBPF‐O/Li cell exhibits high rate capability and good cycling stability. In addition, a flexible LFP/PBPF‐O/graphite battery is successfully fabricated, which can work at stretched, fold, and twisted condition, demonstrating the ability of this electrolyte to be applied in flexible solid‐state devices.

## 4 Experimental Section

### Preparation of Electrolyte Membrane

Poly(vinylidene fluoride‐hexafluoropropylene) (PVDF‐HFP, *M*_W_ = 50 000, Arkema), triethyl phosphate (TEP, 99.5%, Aladdin), fluoroethylene carbonate (FEC, 99%, Aladdin), butyl acrylate (BA, 99%, Aladdin), lithium bis(trifluoromethanesulfonyl)imide (LiTFSI, 99%, Aladdin) (1:3:1:5:1 by mass) were blended and stirred to obtain a uniform slurry. Afterward, poly‐(ethylene glycol) diacrylate (PEGDA, *M*_W_ = 600, 1% of BA by mass), and 2‐hydroxy‐2‐methyl‐1‐phenyl‐1‐propanon (HMPP, 1% of BA by mass) were added into the slurry. Then the mixture was casted on glass followed by an ultraviolet (UV, 1 W cm^−2^) curing for 15 min and the PBPF membrane was obtained.

Poly(ethylene oxide) (PEO, *M*_W_ = 600 000, Aladdin), LiTFSI, ethylene carbonate/Dimethyl carbonate solution (EC/DMC, 1:1 by volume), BA, FEC (1:2.5:9:15:0.2 by mass) were blended and stirred to obtain a uniform slurry. Then PEGDA and HMPP were added in the same proportion as PBPF and the mixed slurry was coated on the PBPF followed by UV curing, after which the double‐layer electrolyte PBPF‐O was obtained. All procedures were performed in an Ar‐filled glove box where the oxygen and moisture were below 0.1 ppm, respectively.

### Assembly of Solid‐State Batteries

The solid‐state coin cell (CR2025) was assembled by laminating LiFePO_4_ (LFP) cathode, PBPF/PBPF‐O membrane, and Li foil successively. The LFP cathode was prepared as follows. LiFePO_4_ powder (MTI), Super P, PVDF‐HFP, LiTFSI (8.5:0.5:0.5:0.5 by mass) were mixed and dispersed in NMP (N‐methyl pyrrolidone) to form a uniform slurry. The slurry was coated on Al foil followed by heated at 60 °C for 12 h to volatilize the NMP. The mass loading of active material on Al foil was about 3.2 mg cm^−2^.

The flexible solid‐state battery was fabricated as follows. PVDF‐HFP, BA, PEGDA (1:5:0.05 by mass) with HMPP (1 wt% of BA) were dissolved in NMP. The slurry was casted on glass followed by UV curing for completely polymerization of BA and PEGDA. This copolymer was used as flexible shell protecting the battery. Afterward, the slurry of PVDF‐HFP, BA, PEGDA, and CNTs (4:20:0.2:1 by mass) with HMPP in NMP was casted on the shell. At the same time, a piece of Al foil was touched with the one side of the slurry. After UV curing, the solidified membrane was coated with the LFP cathode. Next, the composite membrane was heated at 60 °C for 12 h to remove the solvent and the flexible shell/cathode was obtained. The preparation process of electrolyte was depicted in Section 2 and Figure 1. And the graphite coated Cu current collector as the anode was prepared as the same procedures as the cathode. Finally, the cathode, polymer electrolyte, and anode were laminated and the flexible solid‐state battery was finished. All procedures of fabricating solid‐state coin cells and flexible batteries were carried out in the Ar‐filled glove box.

### Material Characterization

Fourier Transform Infrared (FT‐IR) spectra of the solid electrolytes were recorded on an IR spectrometer (Thermo IS50). Thermogravimetric (TGA) curves of electrolytes were tested using a SDT Q600 instrument from room temperature to 800 °C in Ar atmosphere at a heating rate of 10 °C min^−1^. The stress–strain curves of the electrolyte membranes were measured on a Zwick/Roell Z020 universal material testing machine. Scanning electron microscopy (SEM, Hitachi S‐4800) was used to observe the morphology of samples. X‐ray photoelectron spectroscopy (XPS, Thermo Scientific ESCALAB 250Xi) was used to analyze the surface chemistry of samples.

### Electrochemical Characterization

Ionic conductivity of the solid electrolyte was tested using a stainless‐steel (SS) blocking symmetric cell on a Princeton electrochemical workstation over a frequency range from 100 to 0.01 kHz, and calculated from Equation (1)

(1) $$\sigma =\frac{d}{R\cdot S}$$

where *d* is the thickness of the electrolyte membrane, *R* is the bulk resistance of the electrolyte, *S* is the area of the electrode. The electrochemical stability window of the GPE was tested using a SS/GPE/Li cell, which was investigated by linear sweep voltammetry (LSV) from 2.0 to 6.0 V (vs Li/Li^+^) at a scan rate of 0.1 mV s^−1^ on a CHI660D electrochemical workstation (Chenhua Instrument). As for the SS/PBPF‐O/Li cell, the PBPO layer was coated on lithium metal. The Li symmetric cells employing the electrolytes were assembled to evaluate their stability against lithium metal. The Li/PBPF‐O/Li cell was assembled by laminating two PBPF‐O layers back to back to ensure the PBPO layers touched with lithium metal. For comparison, the Li/PBPF/Li cell was assembled using two laminated PBPF layers as well.

The electrochemical impedance spectroscopy (EIS) of the LFP/Li batteries was recorded from 100 to 0.1 kHz on Princeton electrochemical workstation. The cyclic voltammetry (CV) measurements of the LFP/Li batteries were carried out over a potential range from 2.5 to 4.0 V (vs Li/Li^+^) at a scan rate of 0.1 mV s^−1^ on CHI660D electrochemical workstation. The Galvanostatic charge/discharge tests were performed from 2.5 to 4.0 V on a LAND battery testing system at 25 °C.

## Conflict of Interest

The authors declare no conflict of interest.

## Supporting information

Supporting Information
