# Supplementary material for: A Stretchable and Safe Polymer Electrolyte with a Protecting‐Layer Strategy for Solid‐State Lithium Metal Batteries
Source: Adv Sci (Weinh). 2021 May 1;8(15):2003241. doi: 10.1002/advs.202003241 (PMC8336491; doi:10.1002/advs.202003241)
Supplement: Supplementary file 1 — Supporting Information [file ADVS-8-2003241-s001.pdf]

© 2020 Wiley-VCH GmbH

ADVANCED SCIENCE Open Access

Supporting Information

for *Adv. Sci.*, DOI: 10.1002/advs.202003241

A stretchable and safe polymer electrolyte with a protecting-layer strategy for solid-state lithium metal batteries

Shengzhao Zhang, Taibo Liang, Donghuang Wang, Yanjun Xu, Yongliang Cui, Jingru Li, Xiuli Wang, Xinhui Xia, Changdong Gu, and Jiangping Tu**

Supporting Information

A stretchable and safe polymer electrolyte with a protecting-layer strategy for solid-state lithium metal batteries

Shengzhao Zhang, Taibo Liang, Donghuang Wang, Yanjun Xu, Yongliang Cui, Jingru Li, Xiuli Wang, Xinhui Xia, Changdong Gu, Jiangping Tu**

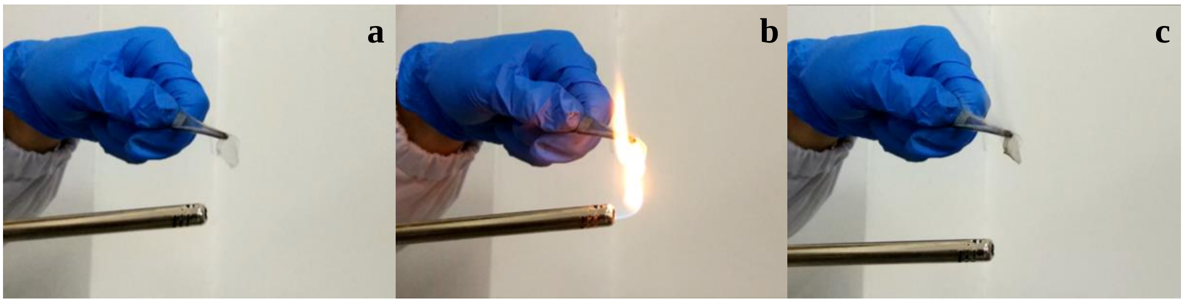

Figure S1. Burning test of PBPF.

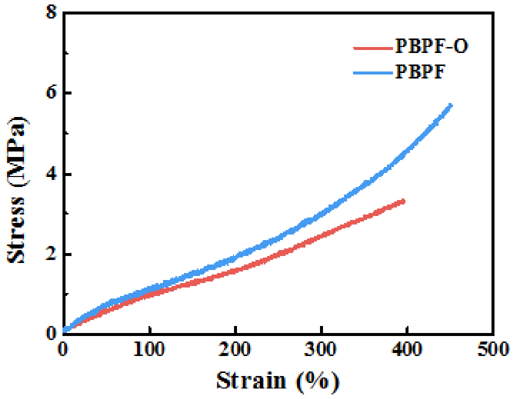

Figure S2. Stress-strain cures of PBPF and PBPO membrane.

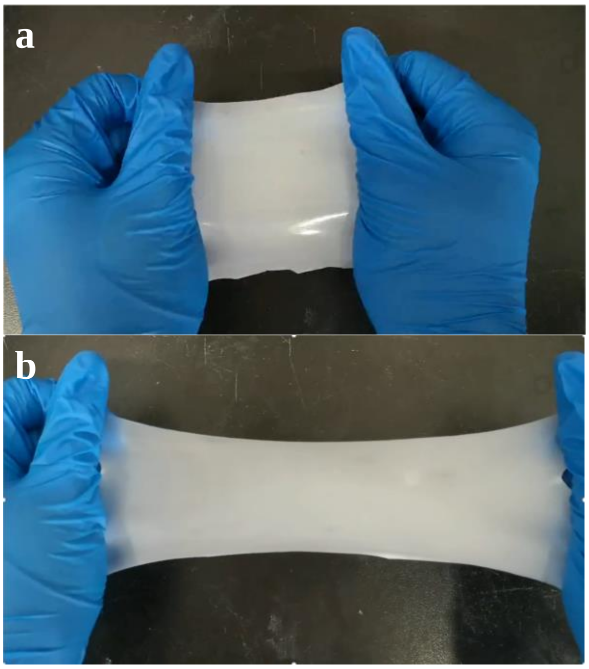

Figure S3. Reality images of PBPF-O membrane at (a) original state and (b) stretched state.

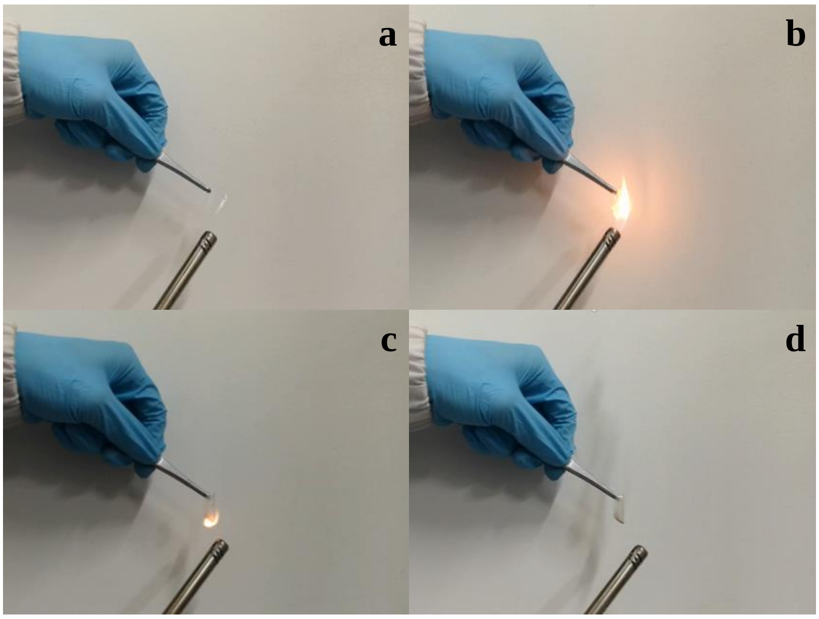

Figure S4. Burning test of PBPF-O.

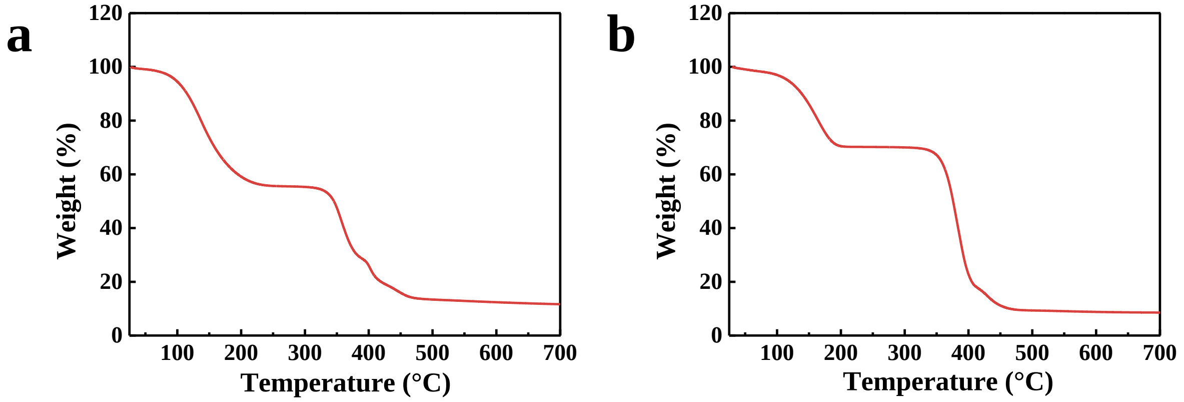

Figure S5. TGA curves of (a) PBPF and (b) PBPO collected from 25 to 700 °C with a heating rate of 10 °C min^{-1} in an Ar atmosphere.

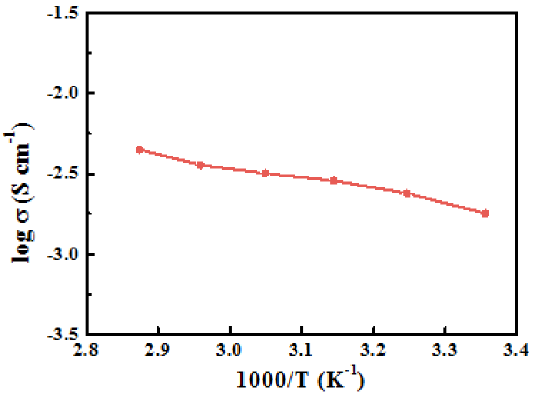

Figure S6. Arrhenius plot showing the temperature-dependent ionic conductivity of PBPF.

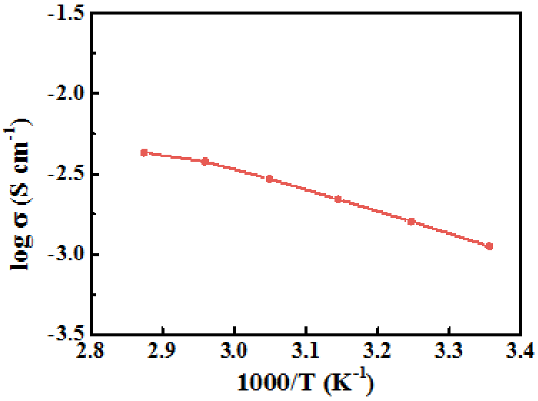

Figure S7. Arrhenius plot showing the temperature-dependent ionic conductivity of PBPO.

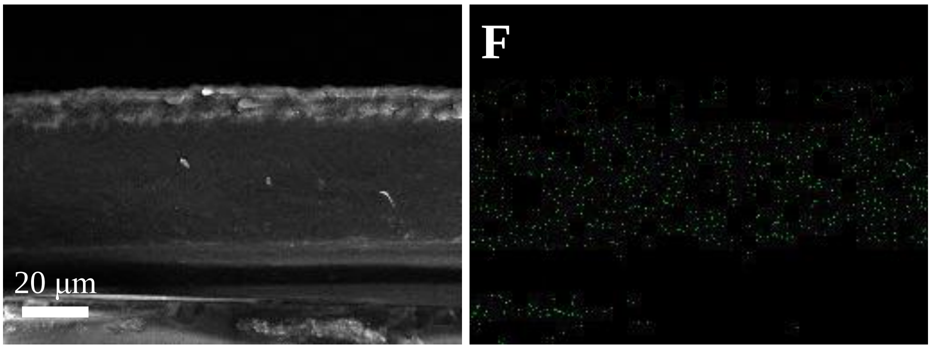

Figure S8. EDX mapping images of F elements in the cross-section of PBPF-O.

The signal of F element in PBPF layer is stronger than PBPO layer due to existence of PVDF-HFP.

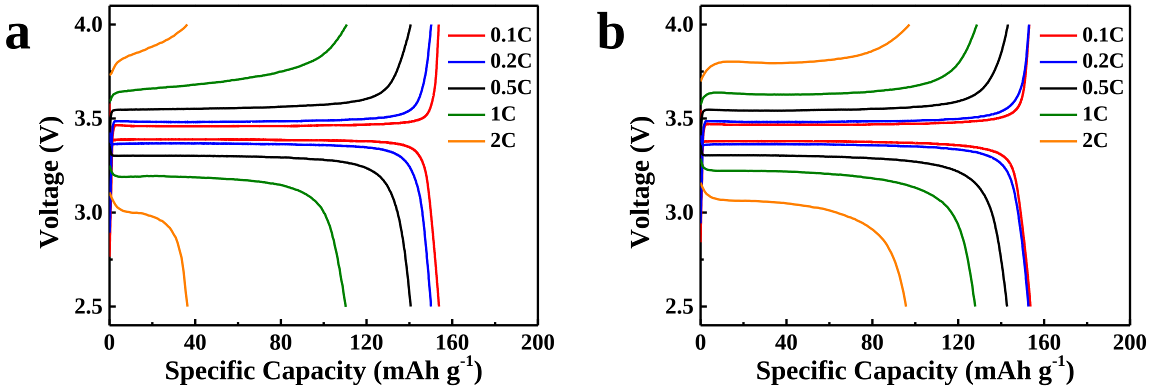

Figure S9. Galvanostatic charge/discharge profiles of (a) LFP/PBPF/Li and (b) LFP/PBPF-O/Li cells at different rates.

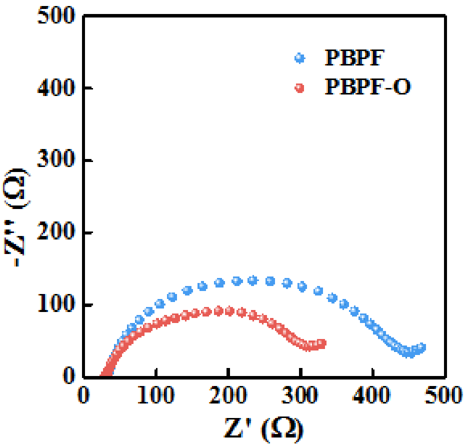

Figure S10. Nyquist plots of LFP/PBPF/Li and LFP/PBPF-O/Li after 100 cycles at 0.5 C.

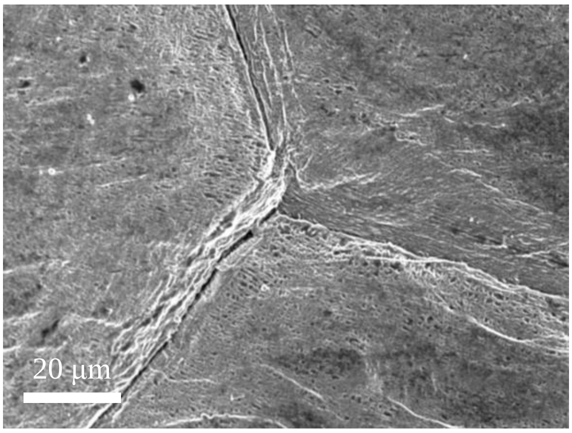

Figure S11. Surface SEM images of lithium anode after 150 cycles at 0.5 C from LFP/PBPF-O/Li cell.

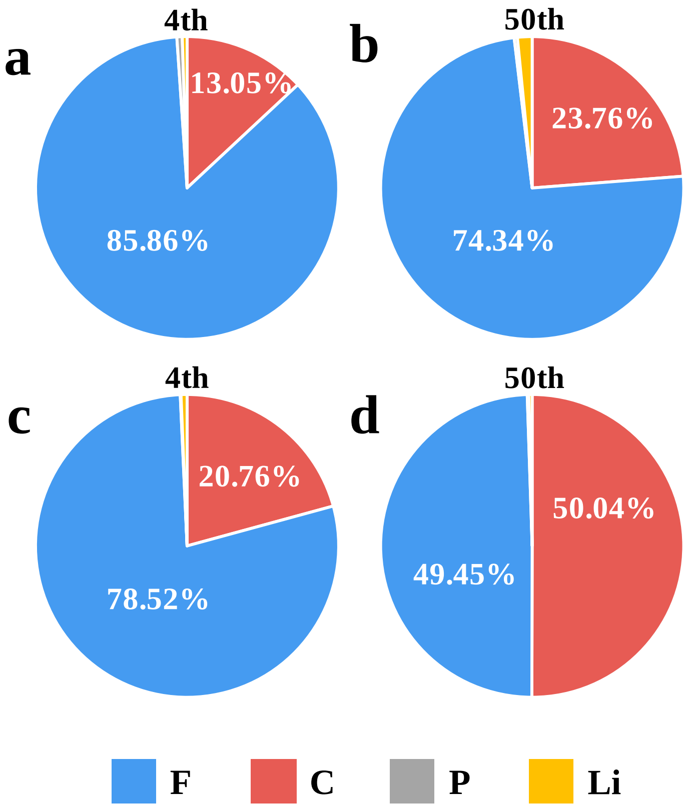

Figure S12. Comparison of elemental proportion in the SEI of (a,b) LFP/PBPF/Li and (c,d) LFP/PBPF-O/Li after different loops at 0.5 C, respectively.
